# Improving information retrieval from electronic health records using dynamic and multi-collaborative filtering

**DOI:** 10.1371/journal.pone.0255467

**Published:** 2021-08-05

**Authors:** Xia Ning, Ziwei Fan, Evan Burgun, Zhiyun Ren, Titus Schleyer

**Affiliations:** 1 Department of Biomedical Informatics, The Ohio State University, Columbus, OH, United States of America; 2 Department of Computer Science and Engineering, The Ohio State University, Columbus, OH, United States of America; 3 Translational Data Analytics Institute, The Ohio State University, Columbus, OH, United States of America; 4 Department of Computer Science, University of Illinois at Chicago, Chicago, IL, United States of America; 5 Defense Finance and Accounting Service, Indianapolis, IN, United States of America; 6 Hyperscience, New York, NY, United States of America; 7 Regenstrief Institute, Indianapolis, IN, United States of America; 8 Indiana University School of Medicine, Indianapolis, IN, United States of America; Vietnam National University, VIET NAM

## Abstract

Due to the rapid growth of information available about individual patients, most physicians suffer from information overload and inefficiencies when they review patient information in health information technology systems. In this paper, we present a novel hybrid dynamic and multi-collaborative filtering method to improve information retrieval from electronic health records. This method recommends relevant information from electronic health records to physicians during patient visits. It models information search dynamics using a Markov model. It also leverages the key idea of collaborative filtering, originating from Recommender Systems, for prioritizing information based on various similarities among physicians, patients and information items. We tested this new method using electronic health record data from the Indiana Network for Patient Care, a large, inter-organizational clinical data repository maintained by the Indiana Health Information Exchange. Our experimental results demonstrated that, for top-5 recommendations, our method was able to correctly predict the information in which physicians were interested in 46.7% of all test cases. For top-1 recommendations, the corresponding figure was 24.7%. In addition, the new method was 22.3% better than the conventional Markov model for top-1 recommendations.

## Introduction

When shoppers consider buying something on Amazon, they often benefit from a section called “Products related to this item.” These recommendations, generated by a method called Collaborative Filtering (*CF*) [1], suggest items of potential interest based on what other customers have viewed and/or purchased. Often, these suggestions are useful and lead to additional purchases: McKinsey has estimated that 35 percent of purchases on Amazon come from these product recommendations [2]. By contrast, when physicians search an electronic health record (EHR) for information about a patient, the EHR does not suggest potentially useful information. Instead, it forces the physician to go through a manual, cumbersome and laborious process of searching for and retrieving information anew for every patient every time.

In this paper, we present a novel method for recommending information items when physicians search for patient information in EHRs. To the best of our knowledge, our method is the first designed to recommend search terms to physicians during patient visits in order to facilitate clinical decision-making.

The literature most relevant to our work addresses Recommender Systems, a research area that originated in computer science. Top-*N* recommender systems, which recommend the top-*N* items that are most likely to be preferred or purchased by users, have been used in a variety of applications in e-commerce. The top-*N* recommendation methods can be broadly classified into two categories [1]. The first is neighborhood-based collaborative filtering methods [3], which recommend items based on the searches of similar users. The second is model-based methods, particularly latent factor models, which learn latent individual user and item factors, and determine user preferences using those factors. Hybrid methods [4] have also been developed to integrate these two types of methods. Recent recommendation methods also include deep learning-based approaches [5], in which user preferences, item characteristics and user-item interactions are learned in deep architectures.

Dynamic recommender systems have been developed to recommend items or information of interest over time. Popular techniques include latent factor transition approaches [6] and Markov models [7] that model the transitions among latent factors that capture preferences; state space approaches [8, 9] that model the transitions among different states over time; and point processes [10] and other statistical models [11] that learn probabilities of future events.

Recently, recommendation methods have also been used to recommend and prioritize healthcare information, due to the rapid growth of information available about individual patients and the need for personalized healthcare [12]. Health recommender systems [13, 14] and recommender systems in health informatics [15] have been applied in several areas. Published work includes recommender systems for tailored health communications [16–18], home medical products [19], personalized recipes [20] and health-related content (e.g., videos, websites, educational materials) [21–23], among others. Additional applications include recommending physicians to patients for specific diseases [24, 25]; medications [26, 27] and therapies [28]; and nursing care plans [29]. However, these studies have primarily used information from sources other than the EHR, such as the Web and published research. The use of recommendations in EHR systems to facilitate clinical decision support has so far been limited.

The main problem with applying recommender systems to EHRs is that, in EHR systems, users do not explicitly rate items as they do on e-commerce and other sites. So, a different mechanism is needed to generate recommendations-specifically, how to identify the next search term for a physician regarding an individual patient. The method we developed to address this need is the ***D***ynamic and ***m***ulti-***C***ollaborative ***F***iltering (*DmCF*) method. The purpose of this paper is to describe the *DmCF* and report the results of our study to test it using EHR data.

The *DmCF* is based on two key ideas: collaborative filtering, which prioritizes items based on the searches of similar physicians regarding similar patients; and dynamic modeling, which predicts items of interest based on how physicians search for information over time. In the name of our method, *dynamic* refers to information retrieval patterns over time, i.e., the order in which different items are searched. (Since searching involves submitting a search term, we use the terms “search term,” “information item,” and “item” interchangeably.) In addition, *Multi-collaborative filtering* (*mCF*) refers to the fact that we integrate multiple types of similarities (e.g., physician similarities, patient similarities and information similarities) to score items of potential interest. The *DmCF* method models information retrieval dynamics using a first-order Markov Chain (*MC*) and combines *MC* transition probabilities with *mCF* scores to produce final scores for items to be recommended. The *DmCF* method then recommends the items with the highest scores to physicians.

In the study reported in this paper, we tested the *DmCF* on a dataset from the Indiana Network for Patient Care (INPC) to determine its effectiveness. We found that the method was successful. For top-1 recommendations (in which only the single highest-scored item is recommended), our results showed that the *DmCF* correctly recommended useful information 22.3% more often than did *MC* models. For top-5 recommendations, the *DmCF* correctly predicted the information in which physicians were interested in 46.7% of all test cases.

Our paper thus makes the following contributions:

- We described our development of the *DmCF*, a novel hybrid dynamic and multi-collaborative filtering method to recommend information items in the EHR to physicians. The *DmCF* combines collaborative filtering (which prioritizes recommended items based on the items similar physicians have searched for with similar patients) with dynamic modeling (which predicts items of interest based on the order in which physicians have searched for items over time).
- We conducted a set of comprehensive experiments using EHR data and demonstrated that the *DmCF* performed significantly better than conventional collaborative filtering-based and Markov-based methods.
- We therefore tackled the problems of identifying and prioritizing the most relevant information items from a large of collection of EHR data to save time and effort for physicians and facilitate their clinical decision-making.

## Methods: Framework of the *DmCF*

This study was approved by the Indiana University IRB (Protocol # 1612682149 “Supporting information retrieval in the ED through collaborative filtering”). In developing the *DmCF*, we wanted a system that would score potential recommended search terms based on combining the following two criteria:

- which terms the physician has already searched for regarding the patient; and
- which terms similar physicians have searched for on similar patients.

The first criterion assumes that past behavior of physicians is a reasonable approximation of the standard of care [30, 31] and that their future behavior follows the same standard of care. Based on this assumption, future search terms can be inferred from previously searched terms and their order. The second criterion considers patient similarities and physician similarities. The underlying concept is that patients have commonalities and that similar patients stimulate similar information retrieval patterns by physicians. Likewise, physicians share traits that result in similar search patterns on patients. For instance, search patterns generated by members of the same medical specialty are likely to resemble each other more than those generated by members of different specialties.

In the description of our method, a physician is denoted as *y*, a patient as *p*, and a search term as *t* (Table 1). A sequence of search terms that a physician *y* searches for on patient *p* during visit *v* is represented as

$$\begin{array}{c} \vec{T}\left(y,p,v\right)=\left\{t_{v_{1}}\to t_{v_{2}}\to \cdots \to t_{v_{k}}|y,p\right\}, \end{array}$$ (1)

where $t_{v_{k}}$ is the *k*-th search term during visit *v*. Additional visits of the same patient with the same physician produce additional search sequences. The physician for whom we recommend search terms is referred to as the *target physician*. The corresponding patient is the *target patient*. A set of physicians/patients similar to the target physician *y*/target patient *p* is denoted as $S_{y}(y)$/$S_{p}(p)$, respectively. A set of search terms similar to a particular search term *t* is denoted as $S_{t}(t)$. Terms are “similar” if they have been searched for on similar patients. Patients are “similar” if physicians search for similar terms regarding them. The size of a set *S* is denoted as |*S*|. Additional symbols will be introduced as they are used.

**Table 1:** Notations.

notation	description
*y* / *p* / *t* / *v*	a physician/patient/term/visit
$\vec{T}(y,p,v)$	a search term sequence of *y* on *p* during visit *v*
$S_{y}(y)$	a set of physicians similar to *y*
$S_{p}(p)$	a set of patients similar to *p*
$S_{t}(t)$	a set of terms similar to *t*

**Fig 1:**
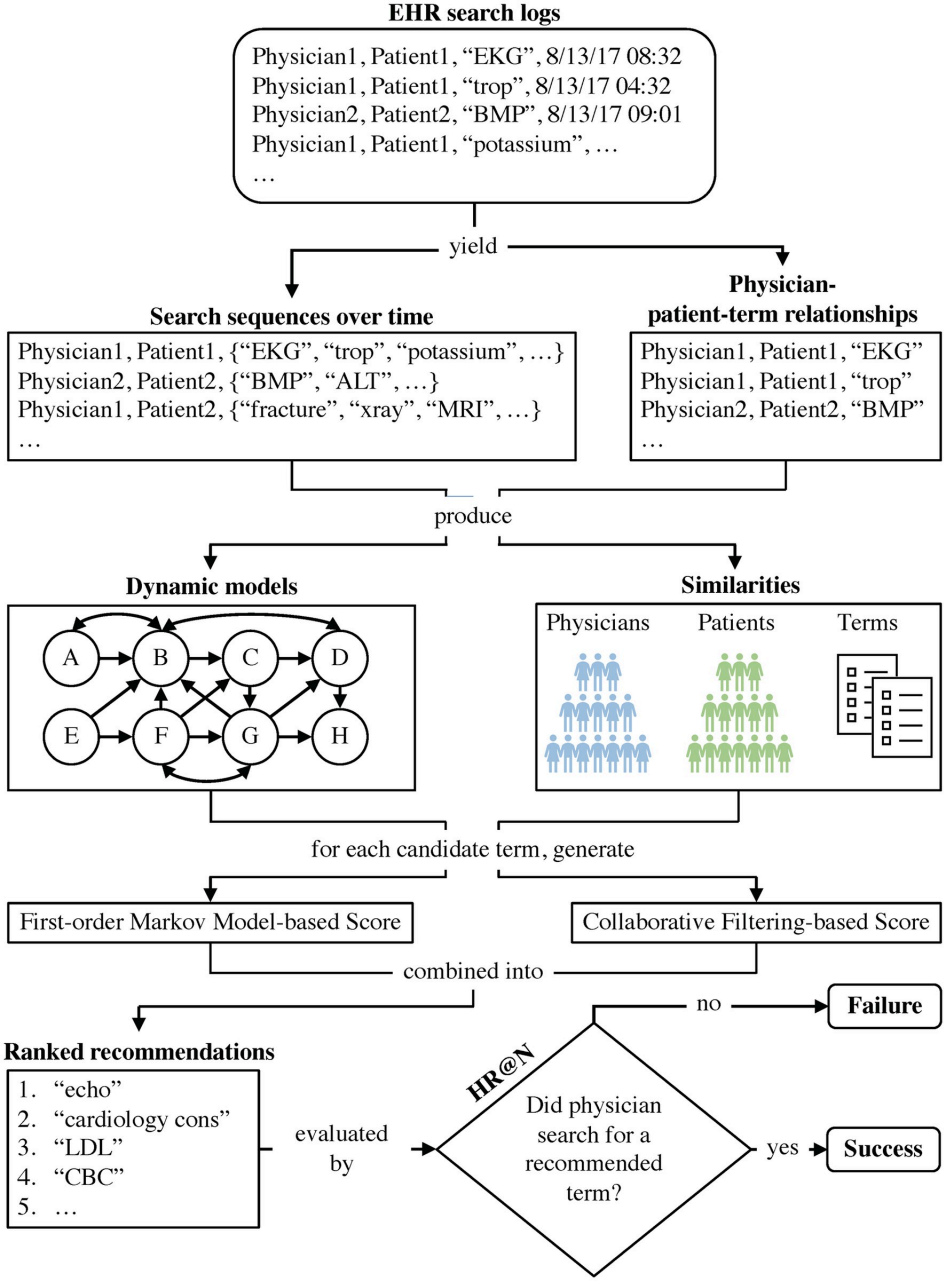
Overall framework of the *DmCF*.

Our *DmCF* method combines search dynamics and multiple similarities to recommend search terms. Fig 1 presents the overall framework of the *DmCF*. The method consists of two scoring components. The first incorporates search dynamics through a first-order Markov Chain [32]. The score of a potential recommendation based on this scoring component is denoted as Score_DYN_. The second component scores search terms based on similarities via multi-collaborative filtering. The score of a potential recommendation based on this similarity-based scoring component is denoted as Score_CF_. Thus, the *DmCF* scores a potential search term *t* for a physician *y* on a patient *p* after a sequence of searches $\vec{T}(y,p,v)$ (Eq 1) as a linear combination of Score_DYN_ and Score_CF_, that is,

$$\begin{array}{c} \begin{array}{cc} & \text{Score}\left(t|\vec{T}\left(y,p,v\right)\right)=\left(1-\alpha \right)\cdot {\text{Score}}_{\text{DYN}}\left(t|\vec{T}\left(y,p,v\right)\right)+\alpha \cdot {\text{Score}}_{\text{CF}}\left(t|\vec{T}\left(y,p,v\right)\right), \end{array} \end{array}$$ (2)

where *α* ∈ [0, 1] is a weighting parameter.

In this paper, if a score is generated from a certain method *X*, a superscript ^*X*^ is included in the score notation (e.g., Score^*X*^, ${\text{Score}}_{\text{DYN}}^{X}$ or ${\text{Score}}_{\text{CF}}^{X}$). In general, a superscript ^*X*^ indicates an associated method *X*. All possible terms are first scored using the scoring function in Eq 2. A higher score represents a higher possibility that a term will be searched next. The terms are sorted based on their scores in decreasing order and the top-*N* (e.g., *N* = 5) scored terms are recommended. If one of the recommended terms is then searched for by the physician receiving the recommendations, the term is considered relevant, and the prioritization of such information is correct. The first-order Markov Chain-based scoring and the multi-collaborative filtering-based scoring are discussed in the next sections. Table 2 lists all methods used in this paper.

**Table 2:** Notations for methods used in the study.

notation	method description
*DmCF*	dynamic and multi-collaborative filtering method
*foMC*	first-order markov chain-based scoring method
*ypCF*	physician-patient-similarity-based *CF* scoring method
*TptCF*	transition-involved patient-term-similarity-based *CF* scoring method
*simP2Y*	patient-first similarity identification
*simY2P*	physician-first similarity identification

### First-order Markov Chain-based scoring—*foMC*

Markov Chains (*MCs*) [32] are a fundamental dynamic modeling scheme based on the Markovian assumption. The Markovian assumption states that each event in a sequence of events (*e*_0_, *e*_1_, *e*_2_, ⋯, *e*_*t*−1_, *e*_*t*_) is only dependent on a small set of previous consecutive events but independent of any earlier events. An *MC* models a sequence of events so that each of the events follows the Markovian assumption. The Markovian assumption is statistically represented as

$$\begin{array}{c} P\left(e_{t}|e_{0},e_{1},e_{2},\cdots ,e_{t-1}\right)=P\left(e_{t}|e_{t-k},\cdots ,e_{t-2},e_{t-1}\right), \end{array}$$

where *P*(*e*_*t*_|*E*) is the probability of observing event *e*_*t*_ given the previous event sequence *E*. The number of previous events that *e*_*t*_ depends on (i.e., *k* in *P*(*e*_*t*_|*e*_*t*−*k*_, ⋯, *e*_*t*−2_, *e*_*t*−1_)) defines the order of the *MC*. A *first-order* *MC* is a special *MC* in which each event depends on only its immediate precursor. *MCs* have been demonstrated to be very effective in modeling, approximating, and analyzing real-life sequences [32].

We use a ***f***irst-***o***rder ***M***arkov ***C***hain (*foMC*) as the dynamic method to simulate the sequence of terms that a physician *y* searches for on a patient *p* during a visit. For a sequence $\vec{T}\left(y,p,v\right)=\left\{t_{v_{1}},t_{v_{2}},\cdots ,t_{v_{k}}|y,p\right\}$, *foMC* calculates a dynamics-based score ${\text{Score}}_{\text{DYN}}^{foMC}$ of a potential search term *t* after $t_{v_{k}}$ as the transition probability from $t_{v_{k}}$ to *t*, that is,

$$\begin{array}{c} {\text{Score}}_{\text{DYN}}^{foMC}\left(t|\vec{T}\left(y,p,v\right)\right)=P\left(t|t_{v_{k}}\right), \end{array}$$ (3)

where $P(t|t_{v_{k}})$ is the transition probability from $t_{v_{k}}$ to *t* in a first-order *MC*. The transition probability *P*(*t*_*j*_|*t*_*i*_) from term *t*_*i*_ to term *t*_*j*_ in a first-order *MC* is calculated as the ratio of the total frequency of transitions from *t*_*i*_ to *t*_*j*_ over the total frequency of all transitions from *t*_*i*_ to any term, that is,

$$\begin{array}{c} P\left(t_{j}|t_{i}\right)=[\sum_{\vec{T}\left(y,p,v\right)}h\left(t_{i}\to t_{j}|\vec{T}\left(y,p,v\right)\right)]/[\sum_{\vec{T}\left(y,p,v\right)}\;\sum_{\left(t_{i}\to t_{k}\right)\in \vec{T}\left(y,p,v\right)}h\left(t_{i}\to t_{k}|\vec{T}\left(y,p,v\right)\right)], \end{array}$$ (4)

where $\left(t_{i}\to t_{k})\in \vec{T}(y,p,v\right)$ represents that (*t*_i_ → *t*_k_) is in $\vec{T}(y,p,v)$, $h(t_{i}\to t_{j}|\vec{T}(y,p,v))$ is the frequency of transitions from *t*_*i*_ to *t*_*j*_ in $\vec{T}(y,p,v)$. Thus, ${\text{Score}}_{\text{DYN}}^{foMC}$ as in Eq 3 is not specific to a particular physician or patient, but corresponds to information retrieval patterns manifesting themselves across all physicians and patients.

### Multi-collaborative filtering-based scoring

Collaborative filtering (*CF*) is a popular technique in Recommender Systems [1] for recommending items to a target user. The fundamental idea of *CF* is that “similar users like similar items.” User-based *CF* methods first identify users similar to the target user, and then recommend items that are preferred by users who are similar to the target user. Item-based *CF* methods first identify items similar to the target user’s preferred items, and then recommend these items to the target user. Thus, *CF* methods heavily depend on the calculation of user similarity and item similarity. A typical way to calculate user similarity is to represent each user using her preference profile over items and to use this item preference profile to identify similar users. Likewise, a typical way to calculate item similarity is to represent each item using its preference profile across users, and use this user preference profile to identify similar items. The user similarity function and item similarity function in *CF* are often pre-defined, and thus recommendations based on similarities can be easily interpreted. *CF* is particularly powerful when user and item data are sparse, which is often the case in real-life applications. *CF* is also well-known for its scalability on large-scale problems, particularly when user similarity and item similarity can be calculated trivially in parallel.

#### Physician-patient-similarity-based CF scoring—*ypCF*

The *CF* method we developed generates search term recommendations based on similar physicians and patients. This method first identifies similar physicians and similar patients, and then scores terms searched by similar physicians on similar patients (each process is described below). This method is referred to as ph***y***sician-***p***atient-similarity-based ***C***ollaborative ***F***iltering (*ypCF*).

*Identifying similar physicians and similar patients*. We developed two approaches to identifying sets of similar physicians and sets of similar patients, depending on which set is identified first: the patients or the physicians. In the first approach, we first identify patients who are similar with respect to the terms that physicians have searched on them. We group these patients into the set of “similar patients.” Then, we assign the physicians who searched for at least one common term between target patient *p* and the set of similar patients to the set of “similar physicians.” In the second approach, we first identify physicians who have searched for common terms across their patients. We group these physicians into the set of “similar physicians.” Then, we identify patients who share at least one search term with the target patient among similar physicians. These patients are then assigned to the set of “similar patients.”

*Patient-first similarity identification*—simP2Y. The first approach first identifies a set of patients similar to the target patient *p*. Based on this set, a set of physicians similar to the target physician *y* is generated. This approach is denoted as *simP2Y*(i.e., from ***P***atients to ph***Y***sicians). In *simP2Y*, the set of patients similar to the target patient *p* is represented as

$$\begin{array}{c} S_{p}^{\text{P2Y}}\left(p\right)=\left\{p_{1},\cdots ,p_{k_{p}}|p\right\}, \end{array}$$ (5)

and is composed of the top-*k*_*p*_ most similar patients to target patient *p*. Given $S_{p}^{\text{P2Y}}\left(p\right)$, a set of physicians similar to the target physician *y* is represented as

$$\begin{array}{c} S_{y}^{\text{P2Y}}\left(y|p\right)=\left\{y_{1},\cdots ,y_{k_{y}}|S_{p}^{\text{P2Y}}\left(p\right)\right\}, \end{array}$$ (6)

and generated as follows: First, physicians who have searched for at least one term on one or more patients in $S_{p}^{\text{P2Y}}\left(p\right)$ that was also searched on *p* are identified. The top-*k*_*y*_ most similar physicians to *y* are then assigned to $S_{y}^{\text{P2Y}}\left(y|p\right)$ (physician-physician similarity will be discussed later in Section Similarity Calculation).

*Physician-first similarity identification*—simY2P. The second approach first identifies a set of physicians similar to the target physician *y*, and then, based on these similar physicians, identifies a set of similar patients. This approach is denoted as *simY2P*(i.e., from ph***Y***sicians to ***P***atients). In *simY2P*, the set of similar physicians is represented as

$$\begin{array}{c} S_{y}^{\text{Y2P}}\left(y\right)=\left\{y_{1},\cdots ,y_{k_{y}}|y\right\}, \end{array}$$ (7)

and includes the top-*k*_*y*_ most similar physicians to *y*. Based on $S_{y}^{\text{Y2P}}\left(y\right)$, a set of patients similar to the target patient *p*, denoted as

$$\begin{array}{c} S_{p}^{\text{Y2P}}\left(p|y\right)=\left\{p_{1},\cdots ,p_{k_{p}}|S_{y}^{\text{Y2P}}\left(y\right)\right\}, \end{array}$$ (8)

is identified as patient *p*’s top-*k*_*p*_ most similar patients on whom physicians in $S_{y}^{\text{Y2P}}\left(y\right)$ have searched for at least one term they have also searched for on *p*.

*Collaborative filtering in* ypCF. From $S_{y}(y)$ and $S_{p}(p)$ (either $S_{p}^{\text{P2Y}}\left(p\right)$ and $S_{y}^{\text{P2Y}}\left(y|p\right)$, or $S_{y}^{\text{Y2P}}\left(y\right)$ and $S_{p}^{\text{Y2P}}\left(p|y\right)$), a set of physician-patient-term triplets, denoted as $S_{ypt}^{ypCF}\left(S_{y}\left(y\right),S_{p}\left(p\right)\right)=\{\langle y_{i},p_{j},t_{k}\rangle |y_{i}\in S_{y}\left(y\right),p_{j}\in S_{p}\left(p\right),t_{k}\in \vec{T}\left(y_{i},p_{j},v_{l}\right),\forall v_{l}\}$, is constructed. That is, $S_{ypt}^{ypCF}\left(S_{y}\left(y\right),S_{p}\left(p\right)\right)$ includes all 〈*y*_*i*_, *p*_*j*_, *t*_*k*_〉 triplets such that physician $y_{i}\in S_{y}\left(y\right)$ has searched for term $t_{k}$ for patient $p_{j}\in S_{p}\left(p\right)$. Thus, for a sequence $\vec{T}\left(y,p,v\right)=\left\{t_{v_{1}},t_{v_{2}},\cdots ,t_{v_{k}}|y,p\right\}$, the score ${\text{Score}}_{\text{CF}}^{ypCF}$ of a potential search term *t* is calculated as follows:

$$\begin{array}{c} \begin{array}{cc} {\text{Score}}_{\text{CF}}^{ypCF} & \left(t|\vec{T}\left(y,p,v\right)\right)=\bar{f}\left(\left\langle y,p,\cdot \right\rangle \right)+\\ & \sum_{\left\langle y^{\prime },p^{\prime },t\right\rangle \in S_{ypt}^{ypCF}}\hat{f}\left(y^{\prime },p^{\prime },t\right)\cdot {\text{sim}}_{y}\left(y,y^{\prime }\right)\cdot {\text{sim}}_{p}\left(p,p^{\prime }\right)/\sum_{\underset{\exists \langle y^{\prime },p^{\prime },t\rangle \in S_{ypt}^{ypCF}}{y^{\prime },p^{\prime }:}}{\text{sim}}_{y}\left(y,y^{\prime }\right)\cdot {\text{sim}}_{p}\left(p,p^{\prime }\right), \end{array} \end{array}$$ (9)

where $\bar{f}(\langle y,p,\cdot \rangle )=\sum_{t:\langle y,p,t\rangle \in S_{ypt}^{ypCF}}f(\langle y,p,t\rangle )/\sum_{t:\langle y,p,t\rangle \in S_{ypt}^{ypCF}}1$ and $\hat{f}(\langle y^{\prime },p^{\prime },t\rangle )=f(\langle y^{\prime },p^{\prime },t\rangle )-\bar{f}(\langle y^{\prime },p^{\prime },\cdot \rangle ),f(\langle y^{\prime },p^{\prime },t\rangle )$ is the frequency of the triplet 〈*y*′, *p*′, *t*〉 (i.e., how many times $y^{\prime }$ searches for *t* on $p^{\prime }$ in total); $\bar{f}\left(\langle y,p,\cdot \rangle \right)$ is the average frequency of all possible terms that *y* searches for on *p*; $\hat{f}\left(\langle y,p,\cdot \rangle \right)$ is the centered frequency for 〈*y*, *p*, ·〉 (i.e., shifted by $\bar{f}\left(\langle y,p,\cdot \rangle \right)$) in order to reduce bias from searches with different frequencies; and sim_*y*_(*y*, *y*′) and sim_*p*_(*p*, *p*′) are the similarity between *y* and *y*′, and the similarity between *p* and *p*′, respectively. The concept behind the scoring scheme in Eq 9 is that the possibility that *y* searches for *t* on *p* after a sequence of searches is the aggregation of 1). the average possibility of *y* searching for any arbitrary search term (i.e., the first term in Eq 9), and 2). the possibility that similar physicians search for *t* on similar patients (i.e., the second term in Eq 9). ${\text{Score}}_{\text{CF}}^{ypCF}$ scores on all possible terms of physician *y* on patient *p* are calculated using Eq 9 and sorted in decreasing order; the top-*N* scored terms are recommended for *y* on *p*.

#### Transition-involved patient-term-similarity-based CF scoring—TptCF

The order in which a physician searches for terms potentially indicates a diagnostic process. Therefore, the search order deserves additional consideration. We developed a new patient-term-similarity-based *CF* scoring method that involves the transitions among search terms. Patient similarities and term similarities are considered in this method, which is different from those in *ypCF*(i.e., physician similarities and patient similarities in *ypCF*). This method is referred to as ***T***ransition-involved ***p***atient-***t***erm-similarity-based ***C***ollaborative ***F***iltering, denoted as *TptCF*.

*TptCF* aggregates the transitions from the last search term in a sequence $\vec{T}(y,p,v)$ (Eq 1) to the next search term for all similar patients. Specifically, *TptCF*identifies a set of patients $S_{p}(p)$ similar to the target patient *p* and a set of terms $S_{t}(t_{v_{k}})$ similar to the last search term $t_{v_{k}}$ in $\vec{T}(y,p,v)$. The set $S_{t}(t_{v_{k}})$ contains the terms with term-term similarity to $t_{v_{k}}$ above a threshold *β*. Then *TptCF* looks into what terms physicians search for on patients in $S_{p}(p)$ after they have searched for a similar term in $S_{t}(t_{v_{k}})$. The underlying assumption is that similar patients stimulate similar patterns of search sequences. Thus, the score ${\text{Score}}_{\text{CF}}^{TptCF}$ of a next potential search term *t* is calculated as follows:

$$\begin{array}{c} \begin{array}{cc} {\text{Score}}_{\text{CF}}^{TptCF} & (t|\vec{T}\left(y,p,v\right))=\\ & \sum_{p^{\prime }\in S_{p}\left(p\right)}\{\frac{{\text{sim}}_{p}(p,p^{\prime })}{\sum_{p^{\prime \prime }\in S_{p}\left(p\right)}{\text{sim}}_{p}\left(p,p^{\prime \prime }\right)}\times \sum_{t^{\prime }\in S_{t}\left(t_{v_{k}}\right)}\frac{g\left(t^{\prime }\to t|p^{\prime }\right){\text{sim}}_{t}\left(t_{v_{k}},t^{\prime }\right)}{\sum_{t^{\prime \prime }\in S_{t}\left(t_{v_{k}}\right)}g\left(t^{\prime \prime }\to t|p^{\prime }\right)}\}, \end{array} \end{array}$$ (10)

where *g*(*t*′ → *t*|*p*′) is the frequency of transitions from term *t*′ to term *t* for patient *p*′ from all possible searches on *p*′, and ${\text{sim}}_{t}(t_{v_{k}},t^{\prime })$ is the term-term similarity between $t_{v_{k}}$ and *t*′. Similarly as in *ypCF*, ${\text{Score}}_{\text{CF}}^{TptCF}$ scores for all possible terms of physician *y* on patient *p* are calculated using Eq 11 and sorted in decreasing order; the top-*N* scored terms are recommended for *y* on *p*.

### Similarity calculation

Key to our method of multi-collaborative filtering-based scoring is to calculate similarities among physicians, patients and terms, respectively. To do so, we represent physicians and patients using vectors of search term frequencies, and terms using vectors of patient frequencies.

**Physician-physician similarities—sim_*y*_**. We first represent each physician *y* using a vector of search term frequencies, denoted as **v**. Each dimension of **v** corresponds to a term, and the value in each dimension of **v** is the total frequency that the corresponding term has been searched by *y*. Note that the frequency is aggregated across all patients that *y* has searched on. This representation scheme is very similar to the bag-of-word representation in text mining [33]. Given the representation, the similarity between two physicians *y* and *y*′ is calculated as the cosine similarity between **v**_*y*_ and **v**_*y*′_, that is,

$$\begin{array}{c} {\text{sim}}_{y}\left(y,y^{\prime }\right)=\cos\left(v_{y},v_{y^{\prime }}\right). \end{array}$$ (11)

The concept is that the search term distribution indicates physician specialty and expertise, and physicians of similar specialties and expertise are considered similar.

**Patient-patient similarities—sim_*p*_**. Similar to physicians, each patient is represented using a vector of term frequencies, denoted as **u**. Each dimension of **u** corresponds to a term, and the value in each dimension of **u** is the total frequency the corresponding term has been searched for by all physicians. The term distribution likely represents the health history of the patient and thus may be a reasonable patient representation. Given that representation, the similarity between two patients *p* and *p*′ is calculated as the cosine similarity between **u**_*p*_ and **u**_*p*′_, that is,

$$\begin{array}{c} {\text{sim}}_{p}\left(p,p^{\prime }\right)=\cos\left(u_{p},u_{p^{\prime }}\right). \end{array}$$ (12)

**Term-term similarities—sim_*t*_**. Each term *t* is represented using a vector of patient frequencies, denoted as **w**. Each dimension in **w** corresponds to a patient, and the value in each dimension of **w** is the total frequency that term *t* is searched for by all physicians. The term-term similarity between terms *t* and *t*′ is calculated as the cosine similarity between **w**_*t*_ and **w**_*t*′_, that is,

$$\begin{array}{c} {\text{sim}}_{t}\left(t,t^{\prime }\right)=\cos\left(w_{t},w_{t^{\prime }}\right). \end{array}$$ (13)

The underlying assumption is that if two terms are frequently searched for on the same patient together, they are either identical or similar in their meaning (i.e., synonymous or closely related), relate to a common medical phenomenon (e.g., “EKG” and “Troponin” can both relate to “myocardial infarction”), or represent co-occurrence of medical concepts (i.e., co-morbidities).

## Methods: Testing the DmCF method

### Data set

The data used to test the *DmCF* method came from the Indiana Network for Patient Care (INPC). The INPC is Indiana’s health information exchange and offers physicians access to the most complete, cross-facility virtual electronic patient records in the nation. Implemented in the 1990s, the INPC collects data from over 140 Indiana hospitals, laboratories, long-term care facilities, and imaging centers. We extracted the INPC search logs generated between 01/24/2013 and 09/24/2013. The Total column in Table 3 summarizes the extracted INPC dataset. Between 01/24/2013 and 09/24/2013, 2,121 physicians performed 69,770 searches on 13,819 patients using 9,781 unique search terms.

**Table 3:** INPC data set used in study: Total and by cutoff periods.

Variable	Total	CUTOFF (06/26/2013)		CUTOFF (07/18/2013)		CUTOFF (08/15/2013)		CUTOFF (09/03/2013)	
		train	test	train	test	train	test	train	test
#*p*	13,819	6,669	587	8,471	624	10,852	472	12,014	372
#*y*	2,121	1,267	126	1,542	147	1,818	126	1,948	105
#*t*	9,781	5,334	665	6,550	654	7,952	532	8,657	461
$\#\vec{T}$	24,183	10,385	648	13,677	692	18,166	535	20,492	414
$\text{len}(\vec{T})$	69,770	28,789	2,568	38,553	2,506	51,272	1,831	58,146	1,482
$\text{len}(\vec{T})/\#p$	5.049	4.317	4.375	4.551	4.016	4.725	3.879	4.840	3.984
$\text{len}(\vec{T})/\#\vec{T}$	2.885	2.772	3.963	2.819	3.621	2.822	3.422	2.837	3.580

#*p* is the number of patients; #*y* is the number of physicians; #*t* is the number of terms; $\#\vec{T}$ is the number of sequences; $\text{len}(\vec{T})$ is total length of sequences; $\text{len}(\vec{T})/\#p$ is average length of sequences per patient; and $\text{len}(\vec{T})\#/\vec{T}$ is average length of sequences. In the CUTOFF columns, train is the number of sequences in the training set, while test is the number of sequences in the test set. The train and test columns do not add up to the total because patients without sequences in the training set were not used for testing. Patients with sequences in the training but not in the test set were used for training, however.

**Fig 2:**
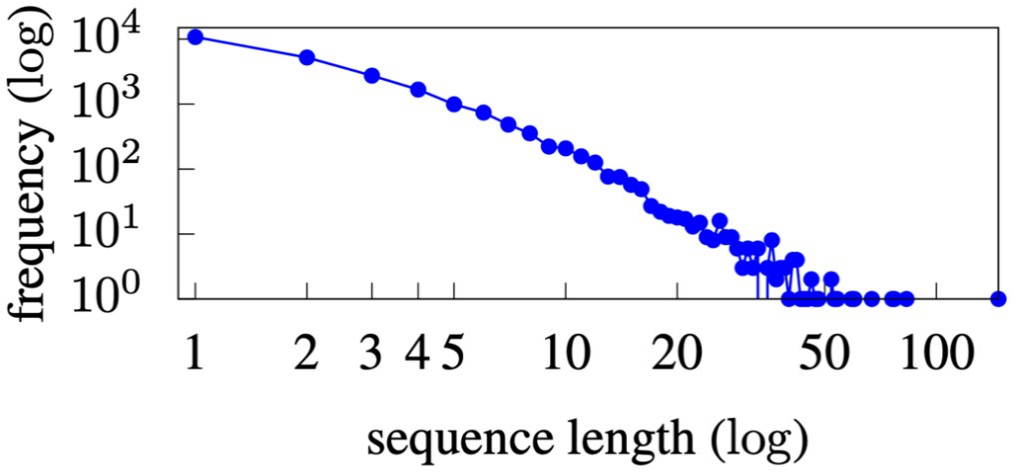
Distribution of INPC sequence length.

**Fig 3:**
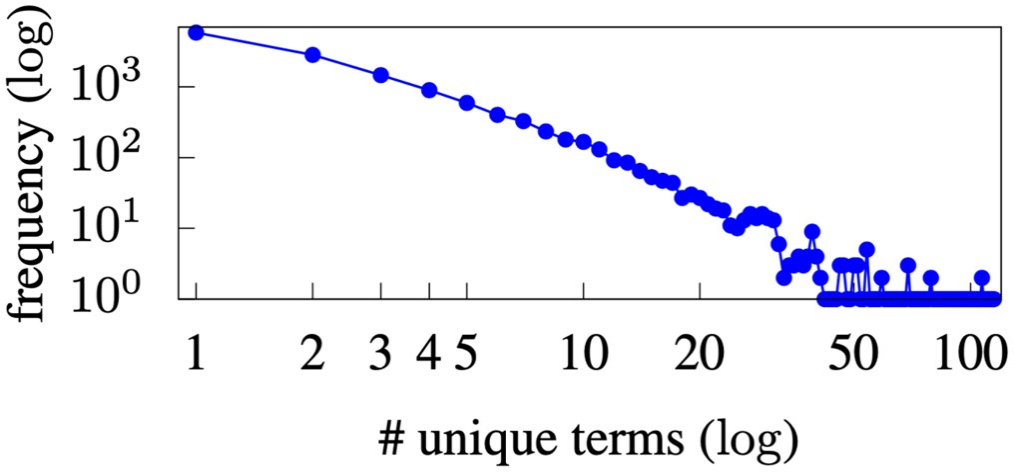
Distribution of INPC # unique terms per patient.

Physicians often conduct multiple, sequential searches on the same patient during a visit, generating a search sequence. Fig 2 presents the distribution of sequence lengths in the dataset. With an average of 2.885 search terms, search sequences were typically very short. Fig 3 presents the distribution of the number of unique search terms for each patient. On average, 3.85 unique search terms were searched for on each patient. The short sequences and small number of unique search terms per patient make the recommendation problem difficult, because the available data are very sparse. It is difficult to learn transition patterns in sequences that are very short. Unfortunately, the data sparsity issue is not unique to the INPC; most EHR systems are not designed to facilitate searches initiated by physicians—they typically display the entire patient record without any prioritization [34–37]. Our method has the advantage of enabling the prioritization of information items that should be displayed instead of all information items.

### Experimental protocols and evaluation metrics

**Fig 4:**
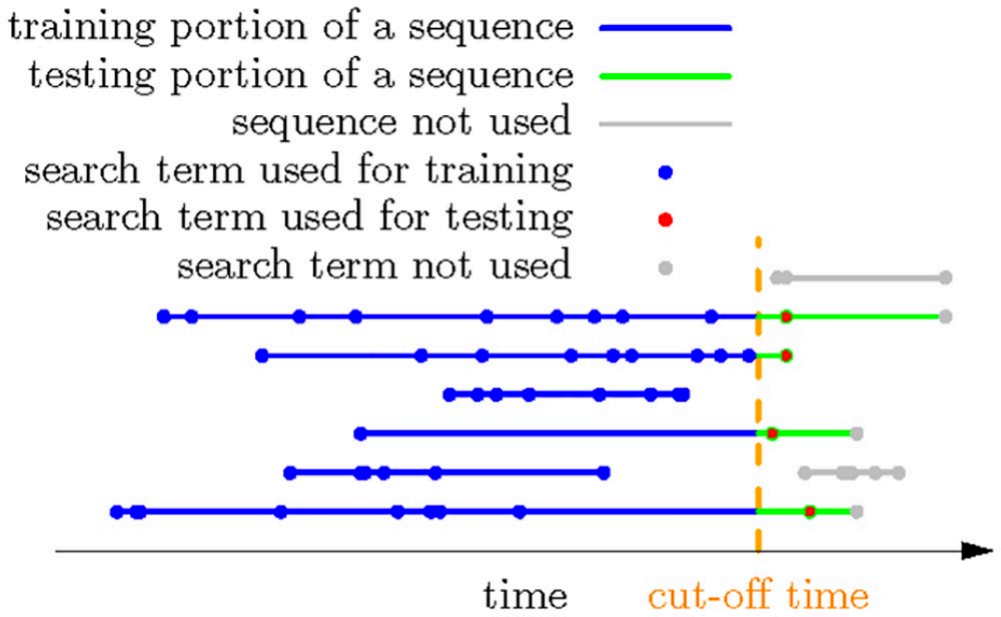
CUTOFF experimental protocol.

We used the following experimental protocol to evaluate our method using the INPC dataset. All search sequences were split at the same cutoff date. All searches before the cutoff date constituted the training set, all searches after the cutoff date the test set. The models were trained using only the training set. For example, the transition probabilities (Eq 4) were constructed only using search sequences and terms in the training set, and the various similarities (Eqs 11, 12 and 13) were calculated only from the training set. This protocol is referred to as cutoff cross validation (CUTOFF). Fig 4 shows the CUTOFF experimental protocol.

We used four cutoff dates (06/26/2013, 07/18/13, 08/15/2013 and 09/03/2013) to generate four sets of training and test data (Table 3) (the time span was specifically 2013-01-24 08:58:26 to 2013-09-24 12:58:32). These cutoff dates were selected to obtain sufficient data for training and testing. We used all search terms involved in calculating similarities. The CUTOFF setting models how we would evaluate performance in the real world where all data up to a certain time point are used to generate a prediction that is then evaluated using the next event. However, a shortcoming of CUTOFF is that if the cutoff date is set early, many late search sequences will not have anything before the cutoff date in the training set; if the cutoff date is set late, many early search sequences may not have terms after the cutoff date as testing terms. Sequences that do not have testing terms are still used to train models. Sequences that do not have training terms are not used in either model training or testing. Thus, the total number of training and testing cases is usually smaller than the entire dataset, as Table 3 shows. For those sequences that had terms after the cutoff date, only the first term after the cutoff date was used for testing and evaluation.

We measured method performance using Hit-Rate at *N* (HR@*N*). *N* is the number of recommended terms. A “hit” occurs when the clinician searches for a term contained in the set of recommended terms. HR@*N* is the percentage of sequences that have a hit. For example, assume that a physician has searched for “CT scan” and “cMRI.” Our method suggests “echocardiography,” “troponin,” and “urine.” If the physician searches for “troponin” next, we have a hit @3 (that is, the term that is searched next is among the top-3 recommendations). The higher the HR@*N* value, the more of the recommendations are correct. The maximum of HR is 1. HR is a popular metric in evaluation of ranking methods [3, 38, 39].

We used Python 2.7.10 to implement all algorithms and conducted all experiments on a Lenovo NeXtScale nx360 M5 server equipped with two 12-core Intel Xeon E5-2680 v3 CPUs and Linux OS.

## Results

### Overall performance

We compared the methods *foMC*, *ypCF*, *TptCF* and *DmCF*, as well as their variations, in our experiments. We performed a grid search to identify the parameters that result in the best performance of each method for five values of HR@*N* (*N* = 1, 2, 3, 4 and 5). Table 4 shows the best performance of each method for the cutoff date 08/15/2013. The best performance of each method with respect to a specific HR@*N* varied given the set of parameters. For example, in Table 4, the row

$$\underline{\bar{\left|DmCF-ypCF\;simP2Y\;0.2\;1\;1\;-\;0.247\;0.357\;\underline{0.426}\;\underline{0.441}\;0.464\;\right|}}$$

shows that the method *DmCF*-*ypCF*, with *simP2Y* as the method to identify similar physicians and *α* = 0.2, $|S_{p}|=1$ and $|S_{y}|=1$, achieved an HR@1 value 0.247, the best HR@1 value this method was able to achieve (therefore, 0.247 is bolded). With the same parameters, this method achieved 0.357, 0.426, 0.441, and 0.464 at HR@2, 3, 4, and 5, respectively. HR@3 and HR@4 outperformed *all* methods with *all* possible parameters (therefore 0.426 and 0.441 are underlined).

**Table 4:** Overall performance of all methods for CUTOFF 8/15/2013.

method	sim	*α*	$\left|S_{p}\right|$	$\left|S_{y}\right|$	*β*	HR@1	HR@2	HR@3	HR@4	HR@5
*foMC*	-	-	-	-	-	**0.202**	**0.297**	**0.338**	**0.378**	**0.393**
*ypCF*	*simP2Y*	-	1	1	-	**0.249**	**0.355**	**0.406**	0.417	0.428
		-	50	2	-	0.215	0.336	0.393	**0.424**	0.441
		-	100	2	-	0.222	0.342	0.393	0.422	**0.443**
	*simY2P*	-	1	1	-	**0.262**	0.292	0.305	0.310	0.320
		-	1	10	-	0.254	**0.329**	0.350	0.368	0.378
		-	2	5	-	0.237	0.312	**0.357**	0.372	0.381
		-	3	20	-	0.230	0.312	0.355	**0.381**	0.393
		-	10	1	-	0.211	0.273	0.336	0.374	**0.398**
*TptCF*	-	-	160	-	0.1	**0.213**	0.279	0.303	0.322	0.331
	-	-	480	-	0.9	0.189	**0.290**	0.320	0.340	0.355
	-	-	480	-	0.1	0.200	0.284	**0.329**	0.355	0.378
	-	-	500	-	0.1	0.200	0.282	0.327	**0.357**	**0.379**
*DmCF*-*ypCF*	*simP2Y*	0.2	1	1	-	**0.247**	0.357	**0.426**	**0.441**	0.464
		0.5	1	1	-	0.245	**0.363**	0.422	0.439	0.464
		0.2	100	2	-	0.226	0.351	0.404	0.430	**0.467**
	*simY2P*	0.5	3	5	-	**0.254**	0.329	0.353	0.379	0.426
		0.1	3	2	-	0.230	**0.346**	0.366	0.402	0.432
		0.1	1	20	-	0.230	0.331	**0.391**	0.424	0.447
		0.1	1	1	-	0.222	0.331	0.383	**0.430**	0.447
		0.2	1	1	-	0.222	0.323	0.378	0.426	**0.449**
*DmCF*-*TptCF*	-	0.8	60	-	0.4	**0.228**	0.307	0.335	0.359	0.379
	-	0.7	40	-	0.1	0.213	**0.312**	0.348	0.376	0.398
	-	0.8	200	-	0.1	0.213	0.303	**0.353**	0.376	0.400
	-	0.6	5	-	0.1	0.209	0.297	0.344	**0.383**	0.406
	-	0.1	1	-	0.1	0.200	0.310	0.346	0.381	**0.413**

The column “sim” corresponds to similarity identification methods; *α* is the weight on CF component in *DmCF*; $\left|S_{p}\right|$ is the number of similar patients; $\left|S_{y}\right|$ is the number of similar physicians; and *β* is the similarity threshold to identify similar terms. The HR (Hit-Rate) columns show percentages of sequences that had a “hit” (a search term in the recommended terms) for 1, 2, 3, 4, and 5 recommended terms. The best performance of each method under each metric is in **bold**. The best overall performance of all methods under each metric is **underlined**.

As an example, for a particular physician *y* who searched for “CT scan” and “cMRI” for a particular patient *p*, *simP2Y* identified one similar patient who was diagnosed with cardiovascular disease and one similar physician who had a major role in caring for this patient. Based on the search history of the similar physician for the similar patient, *DmCF*-*ypCF* recommended 5 terms: “echocardiography,” “troponin,” “cholesterol,” “stroke,” and “lipid panel.” These recommendations are highly related to cardiovascular disease. The ground truth in the test set shows that physician *y* then searched for “troponin.” This resulted in a hit and demonstrated the effectiveness of *DmCF*-*ypCF* in this case.

#### Best method: *DmCF*-*ypCF* with *simP2Y*

Overall, *DmCF*-*ypCF* with *simP2Y* was the best method because it outperformed all other methods on 4 of the 5 performance measures (i.e., HR@2 = 0.363, HR@3 = 0.426, HR@4 = 0.441, and HR@5 = 0.467) (Table 4). With parameters *α* = 0.2, $\left|S_{p}\right|$ = 1 (i.e., with only 1 similar patient) and $|S_{y}|=1$ (i.e., with only 1 similar physician), *DmCF*-*ypCF* with *simP2Y* outperformed the simple *foMC* at 22.3%, 20.2%, 26.0%, 16.7%, and 18.1% on HR@1, HR@2, HR@3, HR@4, and HR@5, respectively. The second best method was *ypCF* with *simP2Y* because it had better overall results than the rest of the methods. With parameters $|S_{p}|=1$ and $|S_{y}|=1$, *ypCF* with *simP2Y* outperformed the simple *foMC* at 22.3%, 20.2%, 26.0%, 16.7%, and 18.1% on HR@1, HR@2, HR@3, HR@4, and HR@5, respectively (e.g., in terms of HR@1, the improvement was 0.247/0.202—1 = 22.3%). It is notable that although *ypCF* was significantly better than *foMC*, the best *DmCF*-*ypCF* with *simP2Y* had a weight *α* = 0.2 on the *ypCF* scoring component, but a larger weight 1-*α* = 0.8 on the *foMC* scoring component, as Eq 2 defines. This indicates the importance of search dynamics in recommending the next search terms. It is also notable that the optimal method (*DmCF*-*ypCF* with *simP2Y*) required only a very small number of similar patients ($S_{p}=1$) and physicians ($S_{y}=1$) to perform well. This demonstrates the effectiveness of *DmCF*-*ypCF* in identifying the most relevant information and leveraging such information for recommendations. Table 5 presents examples of recommendations generated by *DmCF*-*ypCF* with *simP2Y* for 5 clinicians on 5 patients.

**Table 5:** Top-5 recommendation examples generated by *DmCF-ypCF* with *simP2Y*.

training sequence	top-5 recommendations
blood type, rh	**blood bank**, EKG
EKG, BMP, troponin	**hgb**, blood, drug screen, glucose, a1c
EKG, HGBA1C, EF, a1c, HGB, pace	**implant**, EG, cancer, ejection fraction, port
dc, echo, cardiac cath	**cardiology**, troponin, EKG, blood, MRI
cad, echo, nstemi, troponin	**echo**, cardiac, catheter, EKG, blood

Recommendations that the clinician actually searches for, that is, a “hit” are in **bold**.

#### Comparison of *DmCF*-*TptCF* and *DmCF*-*ypCF*

The *DmCF*-*TptCF* method was slightly better than *foMC* (Table 4). With parameters *α* = 0.1, $|S_{p}|=1$ and *β* = 0.1, *DmCF*-*TptCF* outperformed *foMC* at -1.0%, 4.4%, 2.4%, 0.8%, and 5.1% on HR@1, HR@2, HR@3, HR@4, and HR@5, respectively. However, *DmCF*-*TptCF* was significantly worse than *DmCF*-*ypCF* with *simP2Y*. The difference between *DmCF*-*TptCF* and *DmCF*-*ypCF* is that, in *DmCF*-*ypCF*, the similarity-based scoring component (i.e., *ypCF*) does not consider search dynamics and only looks at terms that have been searched by similar physicians on similar patients, regardless of how such search terms transition to the search term of interest, while *TptCF* considers such transitions. The performance difference between *DmCF*-*TptCF* and *DmCF*-*ypCF* indicates that the transition information captured in *TptCF* may overlap with that captured in *foMC* (a component in the *DmCF*). Thus, combining the transition information does not lead to substantial performance gains. On the other hand, the information captured by *ypCF* methods could be complementary to that in *foMC*, and thus integration of *ypCF* and *foMC* in *DmCF-ypCF* resulted in significant performance improvement.

#### Comparison of *simP2Y* and *simY2P*

Within *DmCF*-*ypCF*, *simP2Y* performed slightly better than *simY2P*. The *simP2Y* method first identifies patients similar to the target patient, and then identifies physicians similar to the target physician based on the identified similar patients. The *simY2P* method reverses this order, identifying similar physicians first, then similar patients. The fact that *simP2Y* outperformed *simY2P* in *DmCF*-*ypCF* indicates that when physician search dynamics are considered via *MC*, identifying similar patients is more important than identifying similar physicians. In addition, similar physicians should be identified on the basis of those similar patients. A possible explanation for this observation may be that a more focused and homogeneous group of patients similar to the target patient is critical to complement the *MC* information, as *MC* already considers all patients and all physicians (Eq 4). Another reason could be that since physicians often see many patients with different diseases, high physician similarity may be due to common patients they have but who are different from the target patient. If such physicians are first selected (e.g., in *simY2P*), similar patients identified from these physicians might be very different from the target patient. However, when, as in *ypCF*, information about patients and physicians is not considered, a diverse set of physicians and patients might be beneficial. This may explain why in *ypCF*, *simY2P* actually outperformed *simP2Y* slightly.

#### Comparison of *ypCF* and *TptCF*

When we compare *ypCF* and *TptCF* in Table 4, it is notable that *ypCF* was significantly better than *TptCF*, even though *TptCF* used more patients similar to the target patient (i.e., larger $\left|S_{p}\right|$) to achieve best performance. In *TptCF*, only terms that are similar to the term of interest and also from similar physicians and patients are considered in calculating the scores (Eq 10). However, in *ypCF*, all terms from similar physicians and patients are used. The improved performance of *ypCF* compared to that of *TptCF* may indicate that using more possible terms could benefit recommendations. On the other hand, both *foMC* and *TptCF* consider term transitions, while *TptCF* considers term transitions only among similar terms on similar patients. The experimental results show that *TptCF* performed worse than *foMC*. This may indicate that if term transition is a major factor in determining next search term, transitions from more diverse patients should be integrated.

#### Parameter study

**Fig 5:**
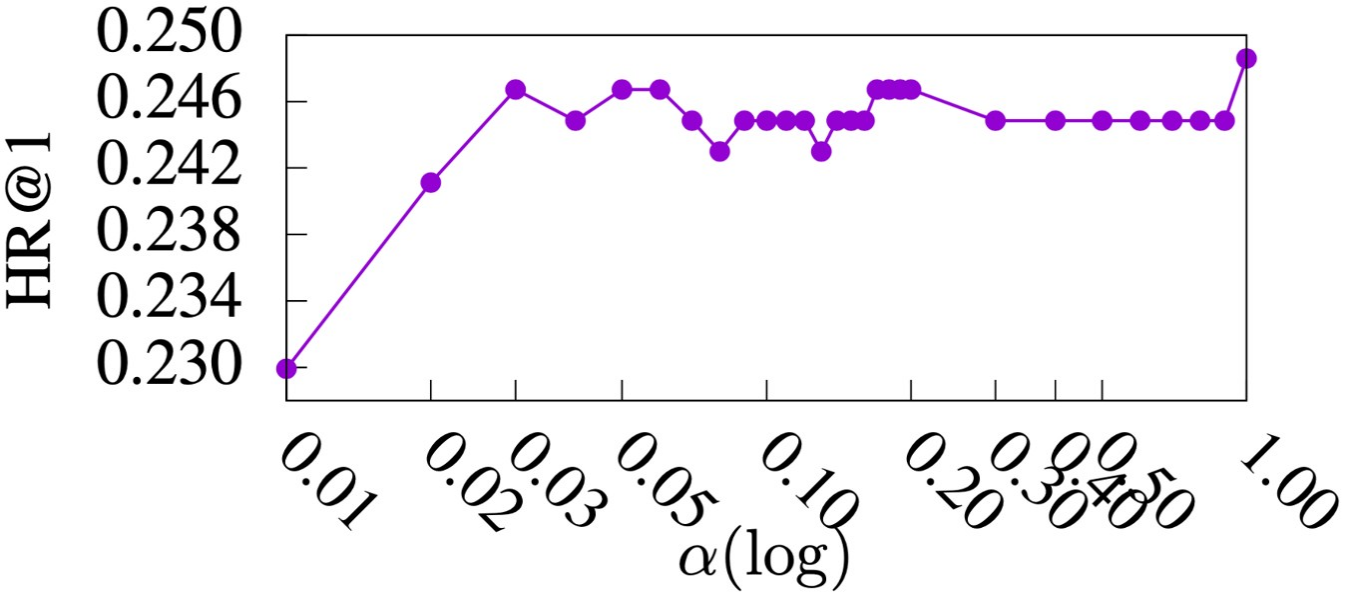
HR@1 over *α* values.

**Fig 6:**
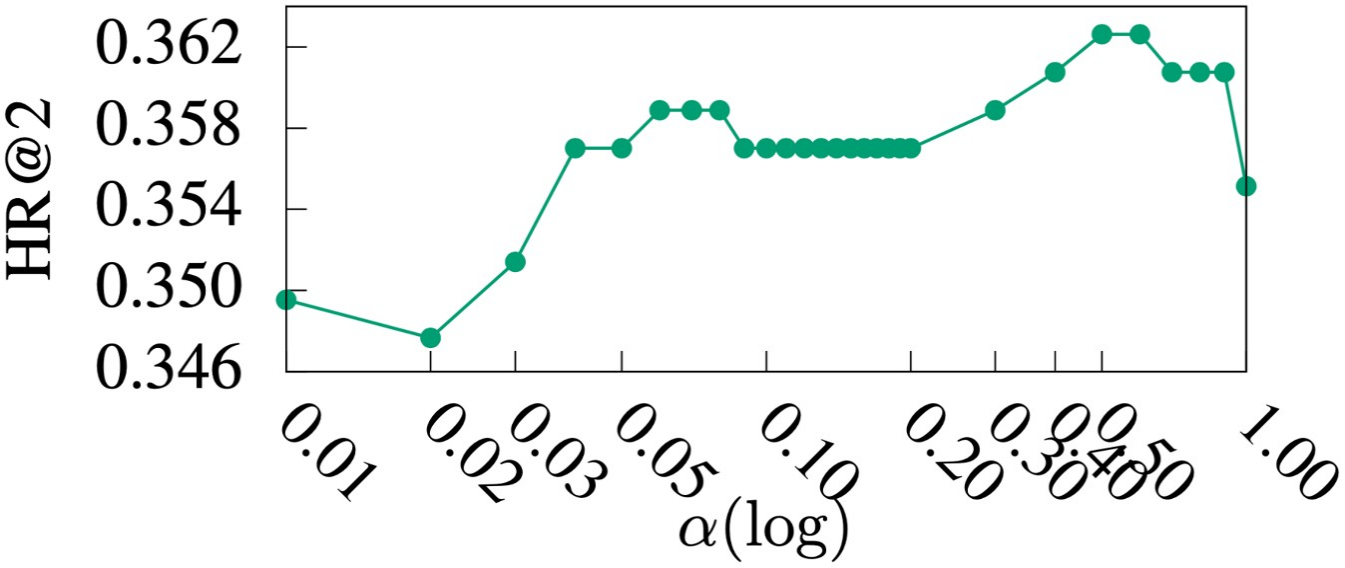
HR@2 over *α* values.

**Fig 7:**
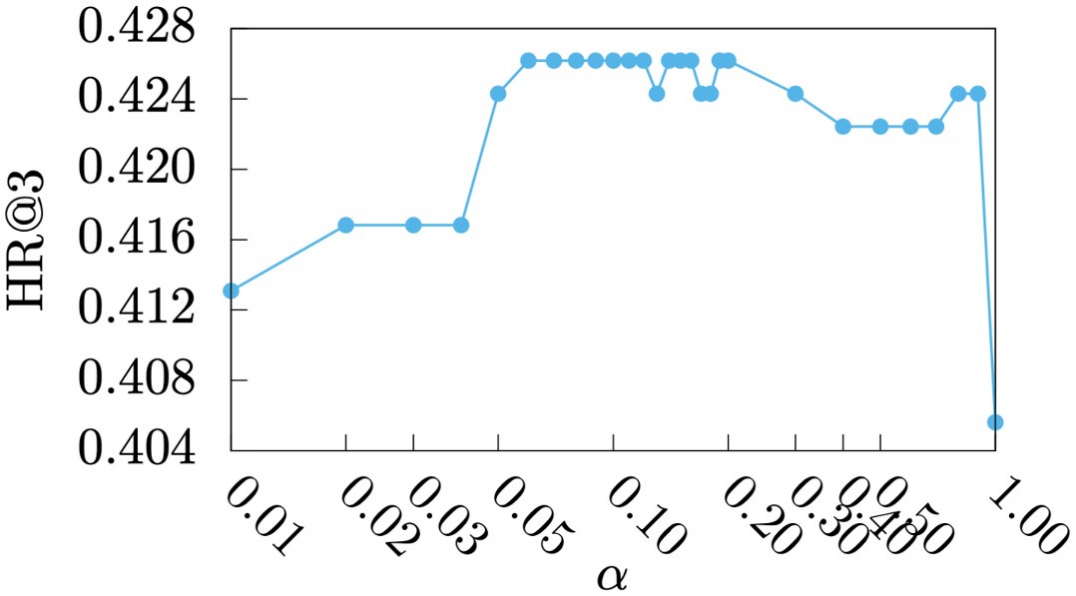
HR@3 over *α* values.

**Fig 8:**
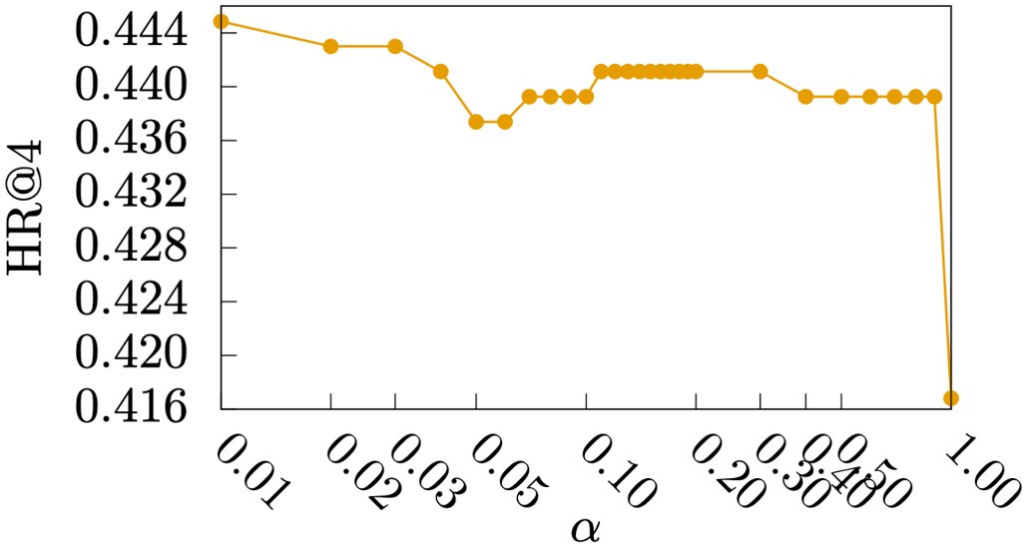
HR@4 over *α* values.

**Fig 9:**
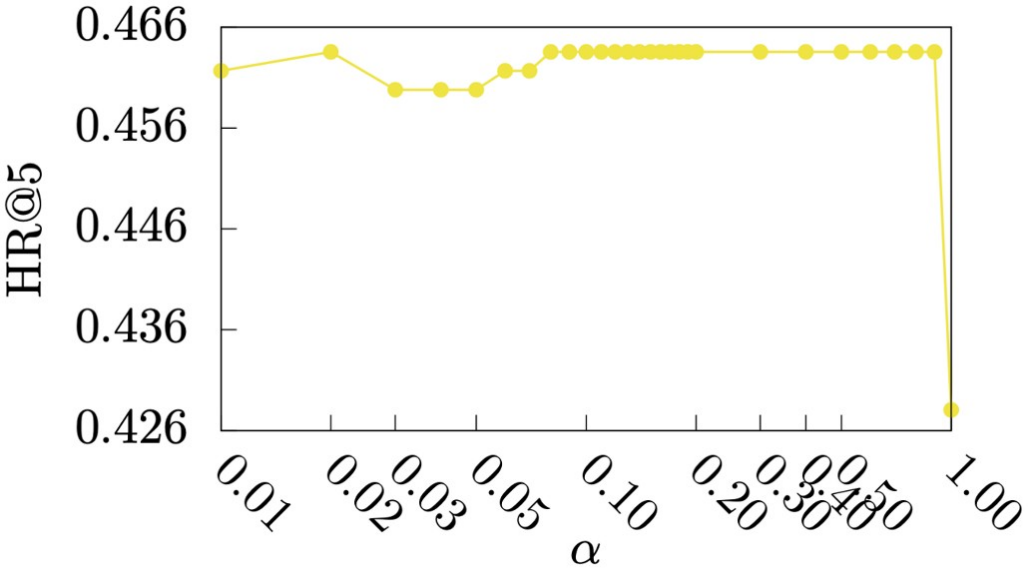
HR@5 over *α* values.

Figs 5–9 show HR@1, HR@2, HR@3, HR@4, and HR@5 of *DmCF*-*ypCF* with *simP2Y* over different *α* values (Eq 2) when $|S_{y}|=1$ and $\left|S_{p}\right|=1$ (i.e., the $\left|S_{y}\right|$ and $\left|S_{p}\right|$ values resulting in the best performance for *DmCF*-*ypCF* with *simP2Y*), respectively. We conducted this analysis to test the effect of *α* parameter and thus the *CF* component on the performance of term scoring (Eq 2). As the weight *α* increased from 0, and, as a result, the *CF* component became more prominent in term scoring, the performance of the *DmCF* in terms of HR@1 and HR@2 generally increased. This demonstrates the effect of the *CF* scoring component in the *DmCF*. As *α* increased further, the performance in general first became better and then worse (except that the HR@1 performance reached its best at *α* = 1). This indicates that the dynamic scoring and *CF* scoring components in the *DmCF* play complementary roles in generating recommendations, and thus combining them results in better recommendation performance than either method alone.

### Overall performance of all methods for other cutoff dates

We also analyzed the performance of all methods for cutoff dates 06/26/2013, 07/18/2013, and 09/03/2013, respectively (Tables 6–8). Overall, *DmCF*-*ypCF* performed best compared to the other methods for various cutoff dates. The comparative performance demonstrated by the different methods for the cutoff date 08/15/2013 remained very similar to that of the other cutoff dates. Note that as more recent cutoff dates increased the size of the training sets (Table 3), the performance of each method decreased. For example, the performance of the *foMC* method decreased progressively as the cutoff progressed. This may be due to the increasing heterogeneity among patients when more patients are available in the system.

**Table 6:** Overall performance of all methods for CUTOFF 06/26/2013.

method	sim	*α*	$\left|S_{p}\right|$	$\left|S_{y}\right|$	*β*	HR@1	HR@2	HR@3	HR@4	HR@5
*foMC*	-	-	-	-	-	**0.205**	**0.313**	**0.341**	**0.369**	**0.381**
*ypCF*	*simP2Y*	-	4	1	-	**0.261**	0.366	0.380	0.383	0.383
		-	50	1	-	0.259	**0.377**	0.398	0.414	0.418
		-	100	1	-	0.250	0.373	**0.403**	**0.418**	**0.431**
	*simY2P*	-	2	3	-	**0.302**	0.350	0.364	0.369	0.372
		-	3	1	-	0.287	**0.370**	0.397	0.414	0.421
		-	5	1	-	0.279	0.360	**0.401**	**0.423**	0.437
		-	10	1	-	0.262	0.349	0.397	0.421	**0.444**
*TptCF*	-	-	200	-	0.1	**0.207**	0.312	0.335	0.347	0.349
	-	-	220	-	0.1	0.204	**0.313**	0.343	0.350	0.353
	-	-	320	-	0.1	0.199	0.313	**0.347**	**0.361**	0.370
	-	-	380	-	0.1	0.194	0.312	0.346	0.356	**0.372**
*DmCF*-*ypCF*	*simP2Y*	0.3	4	1	-	**0.262**	**0.387**	0.415	0.437	0.449
		0.1	20	1	-	0.253	0.377	**0.420**	**0.449**	0.458
		0.2	20	1	-	0.258	0.381	0.420	0.449	**0.460**
	*simY2P*	0.6	3	10	-	**0.262**	0.370	0.407	0.438	0.455
		0.4	3	1	-	0.219	**0.380**	0.409	0.440	0.469
		0.2	3	4	-	0.227	0.375	**0.417**	0.441	0.463
		0.2	2	3	-	0.216	0.363	0.412	**0.451**	0.463
		0.1	5	1	-	0.228	0.373	0.417	0.443	**0.475**
*DmCF*-*TptCF*	-	0.7	5	-	0.1	**0.215**	0.310	0.352	0.381	0.392
	-	0.9	220	-	0.1	0.207	**0.324**	0.356	0.373	0.383
	-	0.8	10	-	0.1	0.208	0.312	**0.360**	0.384	0.394
	-	0.6	10	-	0.1	0.211	0.321	0.355	**0.386**	0.395
	-	0.5	10	-	0.1	0.208	0.318	0.353	0.381	**0.397**

The column “sim” corresponds to similarity identification methods; *α* is the weight on CF component in *DmCF*; $\left|S_{p}\right|$ is the number of similar patients; $\left|S_{y}\right|$ is the number of similar physicians; and *β* is the similarity threshold to identify similar terms. The HR (Hit-Rate) columns show percentages of sequences that had a “hit” (a search term in the recommended terms) for 1, 2, 3, 4, and 5 recommended terms. The best performance of each method under each metric is in **bold**. The best overall performance of all methods under each metric is **underlined**.

**Table 7:** Overall performance of all methods for CUTOFF 07/18/2013.

method	sim	*α*	$\left|S_{p}\right|$	$\left|S_{y}\right|$	*β*	HR@1	HR@2	HR@3	HR@4	HR@5
*foMC*	-	-	-	-	-	**0.210**	**0.292**	**0.325**	**0.341**	**0.348**
*ypCF*	*simP2Y*	-	5	1	-	**0.267**	0.347	0.358	0.364	0.366
		-	50	1	-	0.262	**0.358**	0.379	0.395	0.400
		-	100	1	-	0.257	0.358	**0.384**	**0.402**	0.412
		-	100	2	-	0.237	0.342	0.380	0.396	**0.413**
	*simY2P*	-	2	3	-	**0.289**	0.337	0.353	0.357	0.358
		-	1	100	-	0.283	**0.345**	0.353	0.357	0.358
		-	10	1	-	0.240	0.325	**0.379**	**0.410**	**0.426**
*TptCF*	-	-	260	-	0.1	**0.210**	0.286	0.301	0.312	0.329
	-	-	300	-	0.1	0.207	**0.289**	0.305	0.318	0.329
	-	-	380	-	0.1	0.208	0.288	**0.309**	0.324	**0.341**
	-	-	420	-	0.1	0.208	0.288	0.308	**0.325**	0.340
*DmCF*-*ypCF*	*simP2Y*	0.2	5	1	-	**0.267**	**0.364**	0.393	0.403	0.426
		0.1	50	1	-	0.256	0.355	**0.396**	**0.415**	0.428
		0.2	100	1	-	0.253	0.360	0.396	0.413	**0.431**
	*simY2P*	0.5	2	3	-	**0.251**	0.347	0.387	0.408	0.426
		0.4	2	4	-	0.250	**0.351**	0.392	0.413	0.431
		0.5	5	4	-	0.228	0.341	**0.397**	0.419	0.441
		0.2	5	1	-	0.228	0.335	0.389	**0.423**	0.436
		0.5	10	4	-	0.212	0.315	0.384	0.412	**0.447**
*DmCF*-*TptCF*	-	0.8	5	-	0.1	**0.218**	0.292	0.332	0.351	0.367
	-	0.8	300	-	0.1	0.215	**0.305**	0.328	0.345	0.351
	-	0.6	5	-	0.1	0.217	0.302	**0.340**	0.355	0.364
	-	0.5	5	-	0.1	0.215	0.302	0.338	**0.357**	0.364
	-	0.3	1	-	0.1	0.208	0.292	0.331	0.354	**0.367**

The column “sim” corresponds to similarity identification methods; *α* is the weight on CF component in *DmCF*; $\left|S_{p}\right|$ is the number of similar patients; $\left|S_{y}\right|$ is the number of similar physicians; and *β* is the similarity threshold to identify similar terms. The HR (Hit-Rate) columns show percentages of sequences that had a “hit” (a search term in the recommended terms) for 1, 2, 3, 4, and 5 recommended terms. The best performance of each method under each metric is in **bold**. The best overall performance of all methods under each metric is **underlined**.

**Table 8:** Overall performance of all methods for CUTOFF 09/03/2013.

method	sim	*α*	$\left|S_{p}\right|$	$\left|S_{y}\right|$	*β*	HR@1	HR@2	HR@3	HR@4	HR@5
*foMC*	-	-	-	-	-	**0.193**	**0.271**	**0.304**	**0.331**	**0.365**
*ypCF*	*simP2Y*	-	10	1	-	**0.261**	0.326	0.345	0.355	0.355
		-	20	1	-	0.261	**0.329**	0.353	0.365	0.367
		-	100	1	-	0.246	0.324	**0.374**	**0.399**	**0.406**
	*simY2P*	-	1	1	-	**0.278**	0.329	0.350	0.365	0.365
		-	2	3	-	0.271	**0.336**	0.360	0.379	0.384
		-	10	1	-	0.234	0.304	**0.372**	0.391	0.406
		-	5	1	-	0.242	0.331	0.362	**0.396**	0.408
		-	10	20	-	0.222	0.300	0.360	0.389	**0.413**
*TptCF*	-	-	180	-	0.1	**0.184**	0.246	0.271	0.290	0.304
	-	-	320	-	0.1	0.179	**0.266**	0.295	0.309	0.326
	-	-	500	-	0.1	0.174	0.261	**0.312**	**0.338**	**0.353**
*DmCF*-*ypCF*	*simP2Y*	0.2	10	1	-	**0.263**	0.336	0.377	0.389	0.411
		0.1	10	1	-	0.261	**0.338**	0.377	0.389	0.411
		0.1	100	1	-	0.234	0.331	**0.382**	**0.411**	0.425
		0.2	100	1	-	0.246	0.331	0.382	0.408	**0.428**
	*simY2P*	0.4	3	2	-	**0.242**	0.319	0.355	0.386	0.423
		0.4	2	1	-	0.234	**0.343**	0.384	0.391	0.418
		0.3	3	2	-	0.234	0.336	**0.389**	0.396	0.423
		0.2	4	5	-	0.220	0.333	0.374	**0.403**	0.425
		0.1	2	2	-	0.208	0.312	0.362	0.391	**0.435**
*DmCF*-*TptCF*	-	0.8	40	-	0.1	**0.208**	0.292	0.326	0.348	0.374
	-	0.8	20	-	0.1	0.198	**0.292**	0.321	0.345	0.379
	-	0.9	460	-	0.1	0.181	0.271	**0.338**	0.365	0.382
	-	0.9	480	-	0.1	0.184	0.271	0.333	**0.367**	0.382
	-	0.1	5	-	0.1	0.198	0.278	0.319	0.350	**0.389**

The column “sim” corresponds to similarity identification methods; *α* is the weight on CF component in *DmCF*; $\left|S_{p}\right|$ is the number of similar patients; $\left|S_{y}\right|$ is the number of similar physicians; and *β* is the similarity threshold to identify similar terms. The HR (Hit-Rate) columns show percentages of sequences that had a “hit” (a search term in the recommended terms) for 1, 2, 3, 4, and 5 recommended terms. The best performance of each method under each metric is in **bold**. The best overall performance of all methods under each metric is **underlined**.

### Similarity analysis

**Fig 10:**
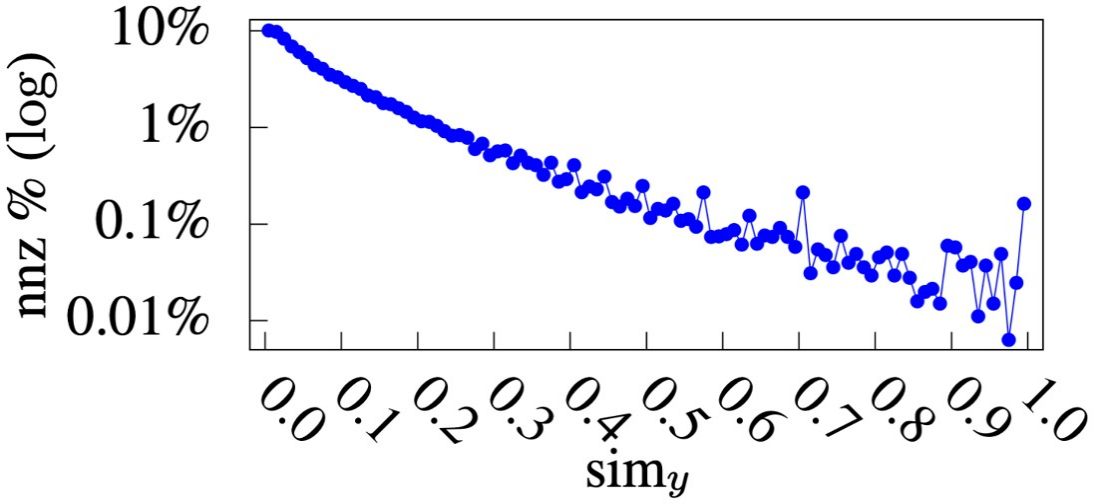
Physician-physician similarity distribution.

**Fig 11:**
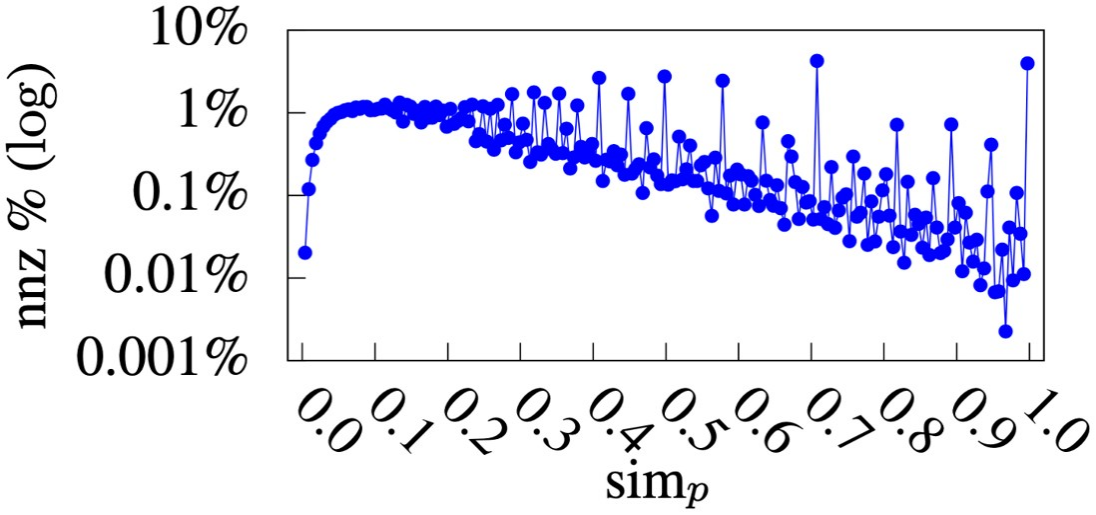
Patient-patient similarity distribution.

Figs 10 and 11 show the distribution of non-zero physician-physician similarities (sim*y*) and patient-patient similarities (sim_*p*_), respectively. For sim*y*, 5.65% of physician-physician similarities were non-zero, and 80.98% of the non-zero similarities were less than or equal to 0.2. For sim_*p*_, 2.65% of the patient-patient similarities were non-zero, and 77.05% of the non-zero similarities were less than or equal to 0.5. Specifically, there were some patients who were very similar to each other (i.e., the peaks in Fig 11 on larger sim_*p*_ values). This explains the advantage of *simP2Y* over *simY2P* (Table 4), because the more patients have a higher sim_*p*_ value in reference to the target patient, the more relevant information the *DmCF* can identify from these patients.

**Fig 12:**
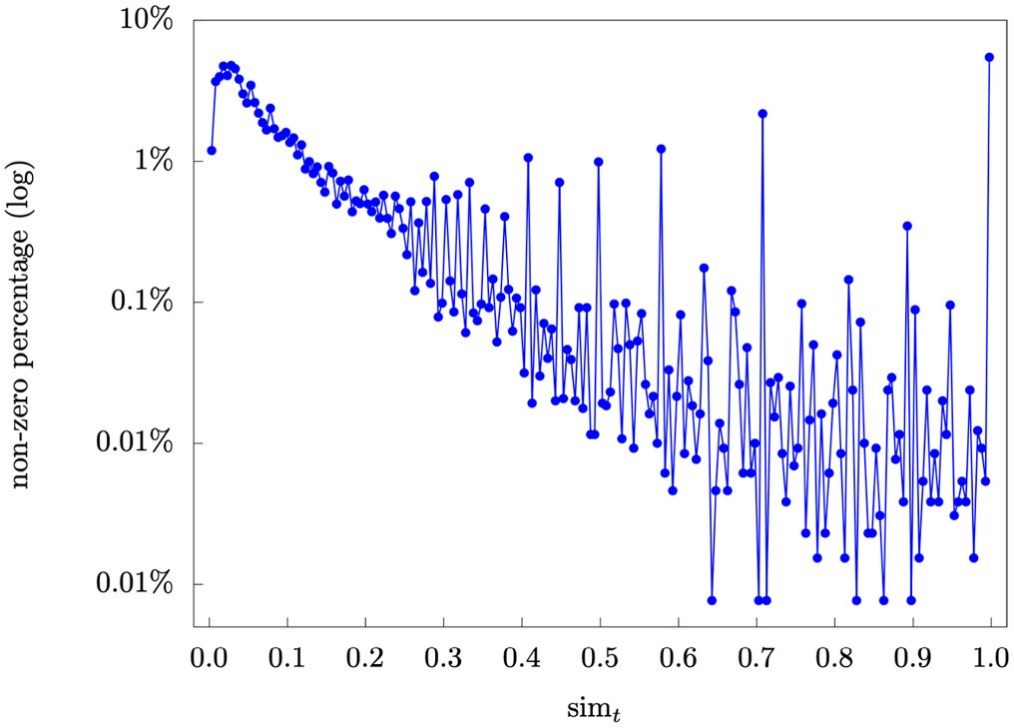
Term-term similarity distribution.

Fig 12 presents the distribution of non-zero term-term similarities (sim*t*). For sim*t*, only 0.28% of term-term similarities are non-zero, and 78.36% of the non-zero similarities are less than or equal to 0.3.

## Discussion

Our method *DmCF* is unique and significantly different from prior recommendation methods in health informatics applications [13, 14]. The major difference is that our methods are designed to recommend items from the EHR for physicians. Thus, our methods can facilitate clinical decision-making in the context of EHR usage and have the potential to directly impact healthcare outcomes. Almost all other published methods are intended for use outside of EHR systems, for example, to recommend healthy diets [20] or health educational content using non-EHR information [23]. While designed with the general purpose of helping improve health behaviors, they are targeted at patients, not healthcare providers. While they can impact health outcomes, they cannot do so by improving clinical decision-making of providers. In addition, our methods use EHR data including clinical variables that much more directly describe patients’ health conditions than website and other information used by other recommendation methods. Thus, our methods can provide tailored and accurate recommendations highly relevant to individual patients. Given that there are no similar methods to those we developed, we have no baseline methods for purposes of comparison. Instead, we implemented *foMC*, *ypCF* and *TptCF* models for comparison. The results reported in Tables 4, 6–8 demonstrate that our new methods significantly outperformed the other methods.

Our method is general and not designed for a particular disease. The reason is that our method is data-driven. When there are similar patients with the same disease in an EHR system, our method is able to identify such patients and their physicians, and calculate recommendations correspondingly. Disease-specific information is implicitly embedded in the patient data, and therefore we do not need to tailor our method to a disease manually. As disease symptoms change over time, the dynamic modeling component of our method can adapt to the emerging information that physicians most recently searched for. In addition, when identifying similar patients or physicians, our method can use the most recent information to find the most similar patients at a specific point in time. In the long term, we will evaluate our method on multiple diseases, particularly those with changing symptoms. To better adapt to different diseases and their dynamics, we will integrate more disease-specific knowledge in the method in the future so the recommendations can be more disease-specific.

In evaluating our method, we did not employ metrics, such as precision, recall, accuracy, area under curve (AUC), and mean squared errors (MSE), that are commonly used to evaluate regression or classification methods. This is primarily because these metrics are not suitable for our recommendation problems. We do not have the ground truth for recommendation scores, and therefore, metrics such as MSE and AUC are not applicable. For Recommender Systems, the top-ranked (e.g., top 5) are far more important than the remaining recommendations. After all, it is not likely that users will click on the 100-th or 1,000-th recommendation. This is particularly true for physicians, who have limited time to review recommendations. Therefore, in healthcare, only the performance of the very top recommendations is a practically meaningful measurement. Thus, unlike in regression and classification problems, precision, recall and accuracy should be calculated only on a few top recommendations to evaluate performance. The Hit-Rate at *N* metric we used is very close to precision and recall in recommendation research. HR@*N* measures precision but only among top-*N* recommendations, not the conventional precision for the entire recommendation list. Physicians search only for a single next term at a time, so there is only one true positive for each instance of a recommendation, and thus recall will have just two possible values: 1 (i.e., a hit) or 0 (i.e., a miss). HR@*N* already encapsulates recall. Accuracy is not suited to our context because high accuracy can be easily achieved by recommending all search terms. However, such recommendations are not useful at all. Hit-Rate is much more tailored to directly evaluating the performance of top-ranked recommendations than regression and classification-based evaluation metrics. It is a very popular metric for Recommender Systems [3, 38, 39]. A detailed discussion on evaluation metrics for ranking and recommendation problems is available in Gunawardana and Shani [40] and Charu [41].

Since we developed the *DmCF*, we have implemented and evaluated an additional method termed ***H***ybrid ***C***ollaborative ***F***iltering ***M***ethod for ***H***ealthcare (*HCFMH*) [42]. *HCFMH* was inspired by the *DmCF* to integrate different collaborative filtering components. It uses more complicated modeling to learn the relations between physicians and terms, and patients and terms, respectively, which may not be observable directly from data. Due to the stronger learning power, *HCFMH* is able to achieve even better performance than that of the *DmCF* in terms HR@*k*. However, the *DmCF* remains very competitive and still outperforms *HCFMH* in terms of HR@1 (i.e., the hit rate at the top-1 recommendations).

To date, we have evaluated our method using historical data and measured how effective it is in recommending items that have been subsequently selected by clinicians. To understand and measure the utility of our method in the real world, it is critical to evaluate it with clinicians in practice. In the near future, we will implement our method in the Web viewer for the INPC. We are currently recruiting clinicians to test and evaluate our method in clinical practice. We will report on this evaluation with users once our study is completed.

In this study, we focused on accurately identifying and prioritizing the most relevant information items among structured data in EHRs. We have not yet leveraged associated information, such as unstructured clinical notes or the biomedical literature, to support prioritization. Using such information may help our recommendations become more targeted and could improve clinical decision-making. Integrating additional information sources would require integration across multiple, heterogeneous information types, such as EHRs, genomics, imaging, and natural language [43, 44]. It would also be necessary to understand the semantics of a search, clinical notes, and literature; conduct causal inference [45]; estimate the relations between an information item and potential evidence; perform uncertain quantification [46]; and characterize the likelihood of the evidence, among many other related issues. Exploiting these opportunities is beyond the scope of this paper, but constitutes important future research that we plan to pursue.

## Conclusion

In this paper, we described and evaluated a new dynamic and multi-collaborative filtering method *DmCF* to recommend search terms for physicians that are relevant to their individual patients. The *DmCF* method combines a dynamic first-order Markov Chain model and a multi-collaborative filtering model in order to score search term recommendations. The collaborative filtering model leverages key ideas developed in Recommender Systems research, and uses patient similarities, physician similarities, and term similarities to score search term recommendation candidates. The findings of our study suggest that the linear combination of dynamic-based and multi-collaborative filtering-based scoring can produce high-quality recommendations that can predict, with top hit rates of approx. 45%, which terms physicians are most interested in. That means that there is an almost 1 in 2 chance that our algorithm correctly predicts the next term.
